# Lutein-Rich Beverage Alleviates Visual Fatigue in the Hyperglycemia Model of Sprague–Dawley Rats

**DOI:** 10.3390/metabo13111110

**Published:** 2023-10-27

**Authors:** Qiong Tang, Sishan Wei, Xiangyi He, Xiaodong Zheng, Fei Tao, Pengcheng Tu, Bei Gao

**Affiliations:** 1 College of Standardization, China Jiliang University, Hangzhou 310018, China; tangqiong@cjlu.edu.cn (Q.T.); 2201501108@cjlu.edu.cn (S.W.); 2101501102@cjlu.edu.cn (X.H.); taofei@cjlu.edu.cn (F.T.); 2 Department of Food Science and Nutrition, Zhejiang University, Hangzhou 310058, China; xdzheng@zju.edu.cn; 3 Department of Environmental Health, Zhejiang Provincial Center for Disease Control and Prevention, Hangzhou 310051, China; 4 Department of Marine Science, School of Marine Sciences, Nanjing University of Information Science and Technology, Nanjing 210044, China

**Keywords:** marigold, lutein-rich beverage, visual fatigue, antioxidant effect

## Abstract

Asthenopia is a syndrome based on the symptoms of eye discomfort that has become a chronic disease that interferes with and harms people’s physical and mental health. Lutein is an internationally recognized “eye nutrient”, and studies have shown that it can protect the retina and relieve visual fatigue. In this study, lutein was extracted from marigold (*Tagetes erecta* L.) and saponified. The purified lutein concentration measured by HPLC was 50.12 mg/100 g. Then, purified lutein was modified to be water-soluble by nanoscale modification and microencapsulation technology. Water-soluble lutein was then mixed with a leaching solution of Chinese wolfberry and chrysanthemum to make a functional beverage. The effects of this beverage on hepatic antioxidant enzymes and the alleviation of visual fatigue in a rat model of diabetes were investigated for 4 weeks. Lutein intake of 0.72 (medium-lutein beverage group) and 1.44 mg/mL (high-lutein beverage group) relieved visual fatigue, ameliorated turbidity symptoms of impaired crystalline lenses, reduced hepatic MDA concentration, increased hepatic GSH concentration, and significantly increased the activities of the hepatic antioxidant enzymes SOD, CAT, GSH-Px, and GR in rats. These data suggest that a lutein-rich beverage is an effective and harmless way to increase the total anti-oxidation capacity of lenses and alleviate visual fatigue.

## 1. Introduction

Current demands of modern society and the popularization of video display terminals have led to long hours of visual task performance in offices, factories, and even at home. People have been overloaded by eye task performance, which can contribute to discomfort in the eyes, the body, and the mind. Likely due to lifestyle changes and the spread of personal computers, increasing rates of such complaints have been reported in various industries [1,2,3].

Visual fatigue, a term derived from the Greek expression “weak eye”, is one consequence that has become a significant public health problem. Symptoms of visual fatigue include impaired reading performance, light sensitivity, blurred vision, diplopia, and perceptual distortions [4,5]. These visual fatigue symptoms can be severe enough to limit personal activities and can potentially accelerate the development of eye diseases such as AMD (Age-related Macular Degeneration), cataracts, diabetic retinopathy, etc. Some epidemiological studies have found that visual fatigue may be associated with systemic symptoms, psychological states, and environmental factors [6,7,8]. Others, however, have reported inconsistent results. Available pharmaceutical treatments for visual fatigue have many limitations, like adverse effects and high rates of secondary failure. Therefore, natural products that can be used to alleviate visual fatigue are currently being investigated [9].

Micronutrients play an important role in the function and health maintenance of the eyes. Specifically, the fat-soluble micronutrient lutein has remarkable functions [10]. Lutein belongs to the xanthophyll family of dietary carotenoids and contains 40 carbon atoms with hydroxylated cyclic structures at both ends. It is abundant in egg yolks and dark green leafy vegetables like spinach and kale [11]. Lutein can also be found in marigold, a member of the Asteraceae family with numerous species. Marigold is an important cultural and well-known ornamental plant that is widespread throughout the world. Marigold has the greatest selection and concentration of lutein derivatives in its flower petals than any other plant [12]. In fact, purified crystalline lutein has a yellow-orange color. Although lutein is not an essential nutrient for human health, its biological activities may be useful in the prevention and reversal of serious eye diseases [13,14].

Despite its ubiquitous presence throughout bodily tissues, lutein and its isomer zeaxanthin are the only carotenoids present in the lens and macular region of the retina. Lutein and zeaxanthin are responsible for central vision and high visual acuity, which suggests that these compounds may play a protective role in these two vital ocular tissues [13,15]. Additionally, lutein is hypothesized to play a similar role in humans as it does in plants. In plants, lutein functions as a potent antioxidant and an effective screener of high-energy blue light. Many epidemiological studies show that high dietary intake of lutein is strongly associated with decreased risk of retinal degenerative diseases, especially AMD and cataracts. Because lutein is a potent antioxidant, it can reduce the risk of degenerative senile plaques in the body and skin. There has also been recent evidence suggesting that lutein may be useful in reducing cancer, and consumption of lutein is thought to play an anti-inflammatory role in maintaining skin health by reducing UV-induced erythema [12,16]. Marigold is considered to be the ideal material for extracting lutein, and it can account for more than 90% of the total carotenoid content because of its high lutein content.

Because humans and primates do not have the capacity for de novo biosynthesis of lutein and zeaxanthin, they depend entirely on dietary sources of these compounds. Today, lutein can be obtained from the diet in several different ways, including supplements and, most recently, functional foods [17]. Functional foods with lutein come in the form of capsules, tablets, and particles [15]. There are few lutein products with beverages as carriers, likely because of lutein’s fat-soluble properties and easy light-oxidization. The effects of a lutein beverage on visual fatigue alleviation have not been studied yet. For our study, we isolated lutein from marigold and modified it into a water-soluble pigment. Then, we mixed it with a leaching solution of traditional plant materials that included Chinese wolfberry and chrysanthemum to develop a functional beverage and investigated its effects on visual fatigue alleviation in rats.

## 2. Materials and Methods

### 2.1. Chemicals

All solvents were HPLC-grade and purchased from Tjshield (Tianjin, China). Lutein (purity 90%) was bought from Hefei Bomei Biotechnology Co., Ltd. (Hefei, China), and streptozotocin (STZ) was purchased from Sigma Co., Ltd. (Saint Louis, MO, USA). Coomassie brilliant blue (CBB) was bought from the Wenzhou Dongsheng chemical reagent factory in Zhejiang province, and bovine serum albumin (BSA) was purchased from Shanghai Juyuan Biotechnology Co., Ltd. (Shanghai, China). Blood glucose test paper was purchased from Roche Diagnostics Products Co., Ltd. (Bangkok, Thailand). MDA, SOD, CAT, GSH, and GSH-PX and GR kits were purchased from Nanjing Jiancheng Bioengineering Institute (Nanjing, China). All the other chemicals were reagent-grade.

### 2.2. Extraction and Saponification of Lutein from Marigold

Samples (500 g) of marigold flowers were accepted from Qijin State, Yunnan Province, China, and they were identified as “Crush” (identified by the Zhejiang Academy of Agricultural Sciences). The samples were ground in a blender, passed through a 0.15 mm sieve, and stored at −20 °C. The extraction and saponification of lutein from dried marigold flower grains were verified. The crude lutein preparation was carried out according to the literature with minor modifications [16,18]. In short, lutein ester hydrolysis was performed in a 500 mL round-bottomed flask connected to a condenser with a boiler. A total of 100 g of the lyophilized marigold petals was added to the flask containing 250 mL of a 10.0 M KOH, 2.5% ascorbic acid solution. The mixture was incubated at 75 °C for 30 min and then cooled to room temperature. The organic layer was mixed with 50 mL of dichloromethane and 20 mL of water and centrifuged at 10,000× *g* for 15 min. Then, the supernatant was collected. This procedure was repeated until the extract was almost colorless, and all extracts were combined. The extract was washed with 30% aqueous ethanol until the water phase was almost colorless and the pH was near neutral. After separation, the organic phase was dried by rotary vaporization at 40 °C. The residue was re-dissolved in 85% aqueous ethanol. The fat-soluble impurities were extracted with hexane. After separation, the concentration of ethanol in the water phase was diluted from 85% to 8.5% using distilled water to precipitate the lutein. The lutein obtained was lyophilized by filtration to yield dry, purified lutein powder. Extracted purified lutein became water-soluble lutein through nanoscale modification and microencapsulation technology. All sample preparations were performed in dim light at room temperature. Careful attention was paid during the extraction and preparation of marigold pigments. The powder was stored at −80 °C until use.

### 2.3. Identification and Quantification of Lutein by HPLC Analysis

The HPLC conditions used to analyze the purified lutein were based on the method in the literature with slight modifications [19]. The HPLC system used was an Ultimate 3000 series with an auto-sampler, quaternary pump system, VDW-3000 detector, thermostatic column compartment, degasser, and Empower software. For separation, an analytical column with 5 μm promosil C18 reversed phase material was used and kept at 25 °C. The mobile phase was acetonitrile, methanol, and water. The flow rate was 1.0 mL/min with a 10 μL injection. Peaks were monitored at 450 nm. Standard solutions of lutein were prepared in dichloromethane and kept in the dark at 4 °C. Working mixtures of pertinent concentrations were prepared daily by appropriate combination and dilution.

All samples were measured under the above-described chromatographic conditions, and quantitative lutein amounts were calculated using a lutein calibration curve. All solvents were pure, and processing was developed in dim light. Samples were analyzed immediately or wrapped in aluminum foil and stored under nitrogen at −20 °C until use.

### 2.4. Preparation of the Lutein Beverage

Currently, no recommended dietary allowances for xanthophyll carotenoids exist in the world. The Third National Health and Nutrition Examination Survey data indicate that Americans consume approximately 2.4 mg/day of combined lutein and zeaxanthin. The recommended allowable daily intake of lutein for the human body is no more than 20 mg/person/day. Based on the probable human body intake of the beverage each day, the beverage’s functional component content was chosen as 1.5 mg/100 mL. To obtain the raw material, a moderate portion of Chinese wolfberry, chrysanthemums, and cassia seed were added to boiling water. This was mixed with lutein, sugar, and citric acid, and the sugar-to-acid ratio was adjusted. The final result was a good-tasting, bright orange beverage. Rats were gavaged with the lutein solution according to Table 1. Lutein doses for the animals were calculated based on human dosages.

### 2.5. Animal Experiments

All experimental procedures were conducted in accordance with protocols approved by the Committee on the Ethics of Animal Experiments of the Science and Technology Department of Zhejiang University and according to the National Institutes of Health Guide for Care and Use of Laboratory Animals. Four week old male Sprague–Dawley (SD) rats weighing 200 ± 15 g were purchased from the National Breeder Center of Rodents and were acclimated for 1 week. Rats were divided into five groups (*n* = 8) that included a high-lutein beverage (HLB) group, a medium-lutein beverage (MLB) group, a low-lutein beverage (LLB) group, a normal group, and a model control group. All groups were allowed free access to water throughout the experiments. Rats were allowed free access to food for 3 days and then fasted for 12–16 h. Afterwards, the normal group was given a single intraperitoneal (IP) injection of citric acid buffer solution. The rest of the groups were given an IP injection of 1% STZ solution at a dose of 65 mg/kg. STZ was administered intraperitoneally in mg/kg body weight. Venous blood was collected from the rats 3 days after injection, and a Roche glucose meter (ACCU-CHEK^®^ Performa, accuracy: 97.6%) was used to measure blood sugar. The rats with blood sugar levels above 9 mmol/L were used for the experimental groups. The HLB, MLB, and LLB groups were gavaged with the beverage according to the weight calculation of 1.44, 0.72, and 0.36 mg/mL daily dose, respectively. The gavage amount was 1 mL per day, and the normal and model groups were gavaged with an equivalent amount of distilled water for 28 consecutive days.

All rats were housed in a room with a temperature of 23 ± 3 °C, 60% humidity, alternating 12 h/12 h light/dark cycle, and ad libitum access to food and water. Body weight, food intake, and water intake were recorded daily. The body weight and blood glucose of the rats were measured and recorded at 3, 15, and 28 days after the STZ injection. After 4 weeks, the rats were sacrificed by decapitation. Liver tissue was collected, weighed, and stored at −80 °C.

### 2.6. Morphological Observation of Rat Lenses

On the 3rd day after STZ injection, rats with a fasting blood glucose value higher than 9 mmol/L had their eyes dilated with tropinamide-dilating eye drops, and the lens morphology of the rats was examined by a hand-held slit lamp, and crystal images were recorded. Twenty-eight days later, the lens shape was dilated again, the crystal images were recorded, and the curative effect was judged according to the requirements of ophthalmology.

### 2.7. Determination of Liver Biochemical Indicators in Rats

The liver samples from each rat were homogenized in PBS and brought to a mass concentration of 10% in a cold, mixed slurry. The MDA and GSH concentrations and the GR, SOD, CAT, and GSH-Px activities were determined by enzymatic methods using commercially available kits from Nanjing Jiancheng Bioengineering Institute. Protein content in the liver was measured using the Coomassie brilliant blue method.

### 2.8. Statistical Analysis

The sample groups were statistically analyzed using SPSS 19.0 statistical software. The mean ± standard error for each group was calculated. Significant differences among groups were examined by single-factor variance. Differences in means were considered significant at *p* < 0.05.

## 3. Results

### 3.1. Identification of Marigold Lutein (ML)

**Figure 1:**
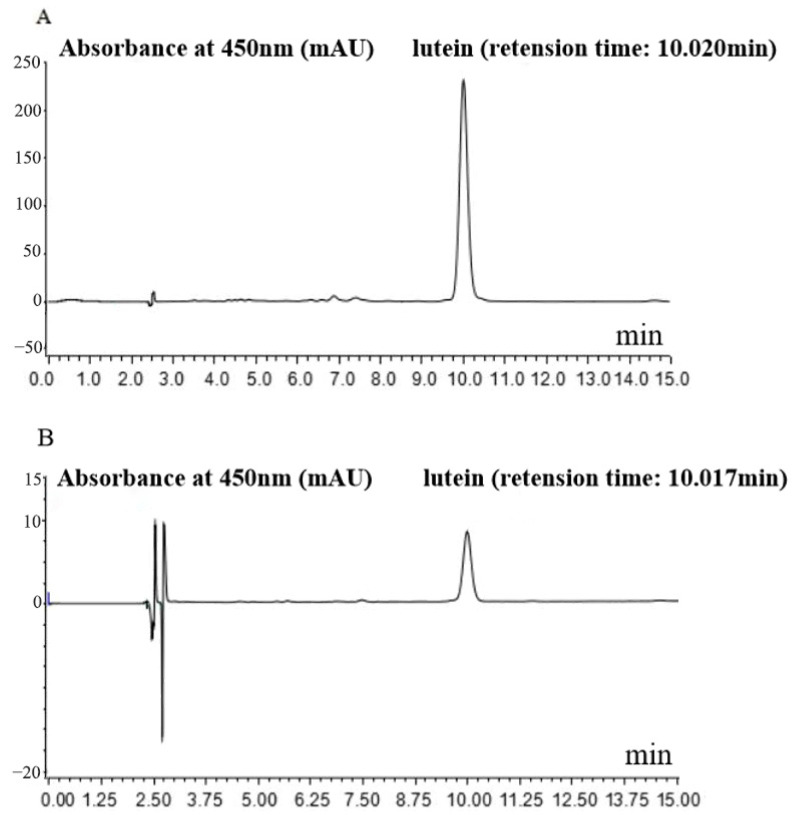
HPLC chromatogram of lutein. (**A**) HPLC chromatogram of lutein standard at 450 nm; (**B**) chromatogram of purified lutein from marigold flowers at 450 nm. Chromatogram conditions: column−Promosil C18 (4.6 mm × 460 nm, 5 μm) at 25 °C; mobile phase—acetonitrile, methanol, and water (83:10:7 *v*/*v*/*v*); and flow rate—1.0 mL/min with 10 μL injection.

The extraction yield of the lutein after saponification was 3.96 mg/g. Then analysis of purified lutein from marigold by HPLC methods was performed in the present study. Figure 1A shows the standard lutein profile at 450 nm by HPLC chromatogram. Purified lutein from marigold was identified (Figure 1B) by comparing the retention time to data previously published and the absorbance spectrum of a lutein standard. The different retention times of lutein we identified may result from climatic conditions, cultivation conditions, varietal diversity, or the influence of the vegetation season. As Figure 1B shows, the retention time of purified lutein was 10.017 min, which was almost the same as the lutein standard. This shows that we were able to isolate relatively pure lutein by saponification. The standard solution of lutein was determined five times. Those results showed that the chromatogram peak area against the injection mass concentration of lutein is linear within the range of 2–50 mg/L. The following is the standard linear regression equation for lutein:

A = 1.3428x − 0.1890 (R^2^ = 0.9999), (1)

x represents the concentration of lutein (μg/mL), and A represents the peak area of the HPLC (mV*min).

Using the lutein standard curve, we calculated the purified lutein concentration from saponified marigold samples as 50.12 mg/100 g.

### 3.2. Effects of Lutein-Rich Beverage on Body Weight and Blood Glucose Level

Throughout the experiment, we observed that rats in the HLB, MLB, and LLB groups exhibited typical symptoms of diabetes 7 days after STZ injection. Compared to the normal group, the normal group and the three lutein beverage groups fed more actively and had whiter and smoother fur than the model group, whose fur was obviously yellow and messy, their bodies were angular, and their activity was not positive.

Table 2 shows the rapid growth of the rats in the normal group, whose weight increased by 34.3% by the end of the experiment. Compared to the normal group, all STZ-induced groups had significantly reduced weight on the seventh day, and throughout the experiment, weight gain was slow, especially in the model group. At the end of the experiment, the weights of the model, HLB, MLB, and LLB groups increased by 12.1%, 18.5%, 16.9%, and 8.8%, respectively.

Table 3 demonstrates that the blood sugar of rats in the normal group remained in the normal range. Three days after STZ injection, a total of 36 rats from other groups had symptoms of hyperglycemia, indicating that the success rate of the model establishment was 90%. Moreover, there were no significant differences in blood sugar levels among the groups after STZ injection, and over the course of the experiment, the levels of each rat group did not significantly change.

### 3.3. Effects of the Lutein-Rich Beverage on the Lens Morphology of Rats

**Figure 2:**
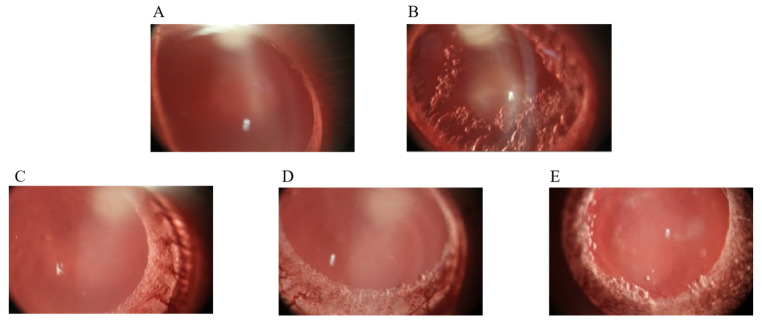
Representative rat lenses from each group. (**A**): Normal group; (**B**): Model group; (**C**): High-dosage group; (**D**): Medium-dosage group; and (**E**): Low-dosage group.

Table 4 shows the effects of the lutein beverage on the crystalline state of diabetic rats. At the beginning of the experiment, the lenses of the rats in each group were wholly transparent. By the end of the test, only the lenses of the normal group remained clear (Figure 2A). The other groups had varying degrees of lens opacity. The nucleus of the lenses in the model group appeared radially white and cloudy, while the nuclear outer cortex had bubble cracks (Figure 2B). Compared to the model group, the groups receiving the lutein-rich beverage had decreased lens opacity. The lenses in the HLB group were as transparent as the normal group (Figure 2C). Small vacuoles were found in some lens nuclei in the MLB group (Figure 2D). Intensive vacuoles and some white opacities were found in some lens nuclei in the LLB group (Figure 2E). It is obvious to see that the lens injuries were alleviated in a dose-dependent manner by the lutein-rich beverage.

### 3.4. Effects of Lutein-Rich Beverage on the Liver Biochemical Indexes in Rats

Table 5 shows that the liver MDA content was significantly increased in the model group compared to the normal group, while the SOD and CAT activities were significantly reduced. The MDA content in the model group was 67.6% higher than the normal group, while the MDA content in the lutein-rich beverage groups clearly decreased. MDA content in the HLB, MLB, and LLB groups was 50.5%, 64.8%, and 81.9% of the model group, respectively. The SOD enzymatic activity of the model group was 50.0% of the normal group, while in the HLB, MLB, and LLB treatment groups it was 79.6%, 72.2%, and 66.7% of the normal group, respectively. SOD enzyme activity was higher in all lutein-rich beverage groups than in the model group. CAT activity in the model group was 56.0% of the normal group. CAT activity decreased in a dose-dependent manner in the HLB, MLB, and LLB treatment groups and was 89.8%, 81.4%, and 70.8% of the normal group, respectively. The enzyme activity had significant reinforcement when compared with the model group.

Table 6 shows that the liver GSH concentration and GSH-Px and GR activity in the model group were significantly lower than the normal group, and lutein-rich beverage treatment had protective effects on GR and GSH-Px enzyme activity. GSH levels were increased 2.00-, 1.67-, and 1.28-fold in the HLB, MLB, and LLB groups, respectively, when compared to the model group. GSH levels in the lutein-rich beverage groups, however, were still well below the normal group. GSH-Px enzyme activity in the HLB, MLB, and LLB groups was 79.4%, 64.0%, and 56.4% of the normal group, respectively, and was higher than the model group. GR enzyme activity in the HLB, MLB, and LLB groups was 37.5%, 25.0%, and 16.7% higher than the model group.

## 4. Discussion

Visual fatigue is a subjective feeling after a long period of visual activity that is a comprehensive embodiment of physiological and psychological fatigue. Visual fatigue is a common problem in modern society, especially among people who need to work on a computer for a long time or look at a screen for a long time. Visual fatigue not only affects an individual’s quality of life but also negatively affects work efficiency. Therefore, reducing visual fatigue is of great significance to people’s lives and work [20,21]. Since the human body is incapable of synthesizing lutein, it must be obtained from foods and dietary supplements. Lutein is present in a wide variety of plant foods and is commonly found in dark green leafy vegetables like spinach and kale. However, people often do not have enough of this important micronutrient in their diet. Because adequate lutein can rarely be achieved by dietary change alone, additional intake in the form of food supplements or functional foods is useful and necessary. Animal toxicology studies have established lutein’s safety as a nutrient and have contributed to purified crystalline lutein’s classification as “generally recognized as safe” (GRAS) [20]. The achievement of GRAS status for purified crystalline lutein highlights the quality and safety of purified lutein and allows for its addition to many foods and beverages. Because they can be supplemented, diets are a viable way to prevent eye-related diseases [22]. A focus on prevention is key because photoreceptors are neural cells that do not undergo mitosis and, once lost, cannot be regenerated.

Many studies have suggested that plant-derived components, including lutein, zeaxanthin, and blackcurrant extract, might have positive effects on visual fatigue recovery in animals. These previous studies thoroughly investigated lutein and visual fatigue and confirmed the beneficial effects of lutein supplementation on visual function [15,17]. However, there are few clinical trials that directly demonstrate that lutein can alleviate visual fatigue. Currently, there are some lutein-containing drugs to treat visual fatigue, but potential side effects prevent their universal use in humans. Furthermore, many people are not aware of visual fatigue because its symptoms are difficult to detect in the beginning. Its development is silent and can lead to more serious eye or physical diseases [2].

For quite some time, research on the effects of water-soluble lutein beverages on the alleviation of visual fatigue has been extremely limited. Almost all the successfully approved visual fatigue alleviation functional foods in China are in the form of capsules, tablets, and particles because of the fat-soluble properties and easy light-oxidization of lutein. In recent years, researchers have begun to focus on the effects of lutein-rich beverages on reducing visual fatigue. Lutein is a natural pigment that exists in plant and animal tissues and has good antioxidant properties. Many studies have shown that lutein can improve visual fatigue symptoms by inhibiting oxidative stress and inflammatory responses. However, the effect of lutein-rich beverages on reducing visual fatigue and the mechanism behind it still need further research. In this study, we developed a lutein-rich beverage with stable properties to ameliorate visual fatigue by modifying fat-soluble lutein into water-soluble lutein. This modification allowed the creation of a more acceptable alleviation supplement for people with visual fatigue pain. The predominant composition of our purified marigold extract was all trans-lutein, which is consistent with previous studies [23,24].

The liver is one of the most important metabolic organs in the human body, with important detoxification and antioxidant functions. The antioxidant enzyme system in the liver includes superoxide dismutase (SOD), glutathione peroxidase (GPx), and glutathione reductase (GR). These antioxidant enzymes can remove free radicals from the body, maintain the REDOX balance in cells, and protect cells from oxidative stress. The occurrence of visual fatigue is related to ocular muscle fatigue, eye dryness, retinal dysfunction, and other factors. Recent studies have shown that oxidative stress is also closely related to the occurrence of visual fatigue [25,26].

In order to study the relationship between changes in liver antioxidant enzymes and visual fatigue, we constructed a Sprague–Dawley hyperglycemia model rat model and administered a lutein-rich beverage to rats to observe its effects on the activity of antioxidant enzymes in rats and its alleviating effect on visual fatigue. We evaluated the changes of antioxidant enzymes by detecting the activity of SOD, GPx, and GR in the rat liver.

Genetic, dietary, and environmental factors affect macular lutein tissue concentrations and trigger visual fatigue and other eye diseases. As expected, our study has confirmed that diabetes mellitus can induce visual fatigue, lens opacity and transparency reduction, and increased hepatic MDA concentrations in rats. Additionally, it decreased hepatic GSH concentrations and SOD, CAT, GSH, GR, and GSH-Px activities [27,28]. When the water-soluble, purified lutein beverage was administered to rats, visual fatigue was attenuated. Hepatic MDA concentrations were decreased; GSH concentrations and SOD, CAT, GSH, GR, and GSH-Px activities were increased. Importantly, the lutein-rich beverage did not cause any abnormal clinical effects during our 4 week experiment.

Lens observations and determination of hepatic MDA and GSH concentrations and SOD, CAT, GSH-Px, and GR activities from our experiment showed the extent of damage to the lenses and livers of rats caused by diabetes mellitus. MDA is a specific marker of oxidative damage to unsaturated fatty acids. Oxidative damage to unsaturated fatty acids directly reflects lipid peroxidation and indirectly reflects the degree of cell damage. GSH is a primary antioxidant in the liver and protects liver proteins from oxidative damage [29,30,31]. In our experiment, the livers of rats in the model group had severe free radical damage when compared to the normal group. Liver MDA concentrations were decreased by 49.5–18.1%, and GSH concentrations were increased by 50.0–33.3% in the lutein-rich beverage treatment groups. These data demonstrate that lutein can reduce free radical damage to the liver and enhance cellular antioxidant capacity. These effects were enhanced by increasing the lutein dose.

SOD is one of the most important antioxidant enzymes that scavenges free radicals in the body. SOD protects the liver from damage by superoxide anion free radicals and derivatives, and it also decreases hepatic free radical concentrations. CAT can eliminate H_2_O_2_ toxicity and reduce the generation of hydroxyl free radicals. GSH-Px is a selenium-containing antioxidant enzyme that widely exists in the body. GSH-Px can eliminate harmful cellular metabolites and block lipid peroxidation chain reactions, playing an important role in protecting normal cell metabolism. Upon diabetes mellitus induction, SOD and CAT are greatly deactivated, likely due to histidine or lysine group modifications [32,33]. When compared to the model group, the SOD, CAT, GSH-Px, and GR activities of the control, HLB, MLB, and LLB treatment groups were increased by 59.3–33.3%, 60.2–26.3%, 66.6–18.4%, and 37.5–16.7%, respectively. These data show that the lutein-rich beverage had protective effects on antioxidant enzymes in the liver. Therefore, lutein can reduce free radical damage to the cells of the eye by protecting the SOD, CAT, GSH-Px, and GR antioxidant enzyme systems, enhancing the regulatory ability of the eye muscles to aid recovery from visual fatigue [34].

The results showed that, compared with the control group, the activities of SOD, GPx, and GR in the liver of rats in the lutein-rich beverage group were significantly increased. This indicates that lutein-rich beverages can increase the activity of antioxidant enzymes in the liver of rats and enhance their antioxidant capacity in vivo. A further study found that the degree of visual fatigue of the rats in the lutein-rich beverage group was significantly reduced, indicating that the lutein-rich beverage could reduce the visual fatigue of the rats. In addition, we also found that the alleviating effect of a lutein-rich beverage on visual fatigue in rats was closely related to the increase in antioxidant enzyme activity. This suggests that the changes in liver antioxidant enzymes may be an important mechanism for the reduction of visual fatigue by lutein-rich beverages.

Lutein in the human body is mainly metabolized by the liver, some of which is converted into other metabolites by the liver and some of which is transported to the eye tissue. The accumulation of lutein in eye tissues is closely related to eye health. Lutein acts as a natural antioxidant, scavenging free radicals and inhibiting oxidative stress. Oxidative stress is one of the important mechanisms of visual fatigue, which can lead to the damage of retinal cells and liver cells. Lutein improves the antioxidant capacity of cells by increasing the activity of antioxidant enzymes such as superoxide dismutase (SOD), glutathione peroxidase (GPx), and glutathione reductase (GR) in the liver and eyes, thereby reducing visual fatigue caused by oxidative stress. Lutein-rich beverages may improve the health of eye tissues by affecting the metabolism and transport of lutein, thereby reducing visual fatigue.

Previous studies have shown that lutein affects the health of the liver and eyes mainly through various ways such as antioxidants, inhibiting inflammation, and promoting energy metabolism, thereby reducing visual fatigue, which is consistent with our study that lutein alleviates visual fatigue mainly by alleviating oxidative stress. This finding provides a theoretical basis for the application of lutein in alleviating visual fatigue, provides a scientific basis for its application in lutein-rich beverages, and provides a new idea for developing other functional foods to promote liver and eye health. However, there are some limitations in the study of the effect of lutein on visual fatigue by establishing a hyperglycemic SD rat model. First, there are differences in lutein metabolism between rats and humans, which means that rats may differ from humans in the bioavailability and metabolic rate of lutein. Second, while visual fatigue occurs in both rats and humans, there may be differences in how it develops and manifests. In rats, visual fatigue is primarily assessed by behavioral observations and physiological indicators. For example, we can assess a rat’s visual fatigue by measuring its reaction time and accuracy in a visual task. At the same time, we can also assess the attention level and brain function status of rats by measuring their pupil diameter and brain electrical activity. However, whether these measurements can accurately reflect the state of visual fatigue in humans remains questionable. Therefore, we need to take into account the potential differences in lutein metabolism and visual fatigue development between rats and humans, and further studies can be conducted to better understand the potential differences in lutein metabolism and visual fatigue development between rats and humans. By comparing the metabolic pathways, lutein bioavailability, and metabolic rate, as well as the development and manifestation of visual fatigue in rats and humans, we can further reveal the limitations of the rat model in studying human visual fatigue and provide a more accurate basis for the interpretation of the findings.

Previous results from diabetic rat models cannot be directly applied to humans because the development of human visual fatigue is influenced by many factors like genetics, diet, environment, etc. The bioavailability of xanthophyll lutein is likely affected by several factors during the various stages of digestion, absorption, and transport processes. Because of this, more studies to elucidate the catabolism and physiological activity of lutein need to be performed. Additional research is also needed to establish the efficacy of the preventive and therapeutic aspects of lutein for certain ocular abnormalities. Large-scale and long-term prospective interventional trials need to be conducted to better understand the role of lutein supplementation in reducing ocular disease risk and alleviating the clinical symptoms of eye disorders in humans. It will also be important to identify the effective daily dosages of lutein and zeaxanthin.

## 5. Conclusions

In summary, our study shows that a lutein-rich beverage alleviates visual fatigue in rats. MLB intake of 0.72 mg/kg ameliorated lens turbidity in rats. Lutein’s alleviation effects were further enhanced with an increased dose. Moreover, lutein-rich beverages drastically decreased the hepatic MDA concentration, increased the hepatic GSH concentration, and significantly increased hepatic SOD, CAT, GSH, GR, and GSH-Px activities. Taken together, these findings may provide insights into the fact that lutein is a safe agent that may be used as a supplement to alleviate the symptoms of visual fatigue.

## Figures and Tables

**Table 1:** The gavage dose scheme of a lutein beverage for rats.

Groups	Lutein Mass Concentration of Beverage/mg/mL	Gavage Volume mL	Lutein Quantity of Gavage mg/kgb	Lutein Quantity in the Human Body/mg/kgb
Normal group	0	1	0	0
Model group	0	1	0	0
High-dosage group	1.44	1	1.44	0.24
Medium-dosage group	0.72	1	0.72	0.12
Low-dosage group	0.36	1	0.36	0.06

Note: Reduced intake of human body (mg/kgb) = gavage amount in rats (mg/kgb)/6.

**Table 2:** Changes in rat weights.

Groups	Average Weight before Test	Average Weight on the 7th Day	Average Weight on the 15th Day	Average Weight on the 28th Day
Normal group	214.9 ± 7.3	234.9 ± 8.1 **	272.8 ± 4.9 **	288.6 ± 6.4 **
Model group	217.1 ± 9.1	207.4 ± 7.2	232.6 ± 12.5	243.4 ± 12.3
High-dosage group	216.1 ± 8.9	204.3 ± 6.4	241.0 ± 24.5	256.1 ± 9.4 *
Medium-dosage group	212.1 ± 12.3	205.0 ± 9.1 *	227.7 ± 24.7	247.9 ± 8.6
Low-dosage group	214.8 ± 8.7	209.8 ± 10.2 *	216.3 ± 28.7 *	233.8 ± 11.1 *

Note: * represents a significant difference of *p* < 0.05 compared to the model group; ** represents a significant difference of *p* < 0.01 compared to the model group.

**Table 3:** Construction of a hyperglycemic rat model.

Groups	Blood Sugar Content/mmol/L on the 3rd Day	Blood Sugar Content/mmol/L on the 15th Day	Blood Sugar Content/mmol/L on the 28th Day
Normal group	3.81 ± 0.26	3.85 ± 0.18	3.76 ± 0.21
Model group	15.06 ± 2.26 **	14.93 ± 1.98 **	14.68 ± 2.29 **
High-dosage group	13.06 ± 2.62 **	13.46 ± 3.12 **	13.91 ± 4.15 **
Medium-dosage group	13.88 ± 2.17 **	11.95 ± 3.47 **	10.23 ± 4.67 **
Low-dosage group	14.30 ± 1.50 **	13.87 ± 2.18 **	13.65 ± 2.04 **

Note: ** represents a significant difference of *p* < 0.01 compared to the model group.

**Table 4:** The effects of the lutein beverage on the crystalline state of diabetic rats.

Groups	Sample Number	Crystalline State		Positive Rate (%)
		Positive	Negative	
Normal group	8	0	8	0 **
Model group	8	6	2	75
High-dosage group	8	1	7	12.5 *
Medium-dosage group	8	2	6	25
Low-dosage group	8	4	4	50

Note: * represents a significant difference of *p* < 0.05 compared to the model group; ** represents a significant difference of *p* < 0.01 compared to the model group.

**Table 5:** The effects of the lutein beverage on the content of MDA and activity of SOD and CAT in the liver tissue of rats in each group.

Groups	MDA (nmol/mg Protein)	SOD (U/mg Protein)	CAT (U/g Protein)
Normal group	10.4 ± 1.49 **	5.4 ± 0.4 **	48.9 ± 2.3 **
Model group	32.1 ± 2.5	2.7 ± 0.6	27.4 ± 2.2
High-dosage group	16.2 ± 1.6 **	4.3 ± 0.3 **	43.9 ± 2.6 **
Medium-dosage group	20.8 ± 1.6 **	3.9 ± 0.7 **	39.8 ± 1.9 *
Low-dosage group	26.3 ± 1.8 *	3.6 ± 0.6 *	34.6 ± 1.9 *

Note: * represents a significant difference of *p* < 0.05 compared to the model group; ** represents a significant difference of *p* < 0.01 compared to the model group.

**Table 6:** The comparison of liver GSH concentration and GR and GSH-Px activities in rats.

Groups	GSH (mol/g Protein)	GR (U/g Protein)	GSH-Px (U/mg Protein)
Normal group	2.1 ± 0.2 **	3.9 ± 0.1 **	68.4 ± 3.7 **
Model group	0.9 ± 0.1	2.4 ± 0.2	32.6 ± 3.0
High-dosage group	1.8 ± 0.1 **	3.3 ± 0.4 **	54.3 ± 2.9 **
Medium-dosage group	1.5 ± 0.12 **	3.0 ± 0.1 *	43.8 ± 4.1 **
Low-dosage group	1.2 ± 0.05 *	2.8 ± 0.1 *	38.6 ± 2.9 *

Note: * represents a significant difference of *p* < 0.05 compared to the model group; ** represents a significant difference of *p* < 0.01 compared to the model group.

## Data Availability

The data presented in this study are available on request from the corresponding author. The data are not publicly available due to copyright.
